# BDryFish: A smartphone image dataset of dried fish (Shutki) trade categories from Bangladeshi markets

**DOI:** 10.1016/j.dib.2026.113359

**Published:** 2026-10-21

**Authors:** Tanvir Anjum Apurbo, Kh Faysal Ahammed, Md. Abdullah, Mohosinin Islam, Md. Habibullah Belali

**Affiliations:** Department of Computer Science & Engineering, Southeast University, 252, Tejgaon Industrial Area, Dhaka, 1208, Bangladesh

**Keywords:** Dried fish, Shutki, Market trade categories, Image classification, Fine grained recognition, Data leakage, Food authentication

## Abstract

Dried fish, known in Bangladesh as shutki, is traded under local names that do not correspond neatly to biological species. BDryFish contains 5778 photographs of 22 dried fish trade categories, taken with three smartphones, with and without flash, on 14 and 21 May 2025 at two wholesale markets in Dhaka, Karwan Bazar and Rayerbazar Shutki Arot. Every category is shown as isolated specimens (Single, 2981 images) and as market heaps (Bulk, 2797 images). The labels are the Bangla trade names given by sellers, so the label space combines species with processing state and cut. English and scientific names were matched from online sources and are not independent taxonomic identifications. Two pairs of classes share a species (whole and sliced hilsa, sun dried and fermented swamp barb), two hairtail classes are size grades, Chouka has no established scientific identity, and one class is a crustacean. Images are released at full resolution and at 640 pixels on the long side, without padding. A metadata table records capture time, device, lens, market, flash state and capture session identifiers at four time thresholds. Because frames were taken in bursts, a random split would put near identical frames in training and test sets, so the recommended split keeps whole capture sessions together. The dataset supports image classification benchmarks under market conditions, transfer between isolated and heaped presentations, shifts across devices and capture days, and recognition of processing states for food authentication. It is an image classification resource; object detection would require additional annotation.

Specifications Table

**Table:** 

Subject	Computer Sciences
Specific subject area	Image classification of dried fish (shutki) trade categories sold in Bangladeshi wholesale markets, for computer vision and food authentication research
Type of data	Image (JPEG), at retained full resolution and at 640 px on the long side. Table (CSV) of acquisition metadata, capture session identifiers and a recommended data split for every image, with SHA-256 checksums.
Data collection	Images were taken on 14 and 21 May 2025 at two wholesale dried fish markets in Dhaka, Bangladesh. At Karwan Bazar, 509 images of two classes were taken at the stalls of two vendors on 14 May. All other images were taken at Rayerbazar Shutki Arot, where every stall, roughly 24 in all, was visited and each variety not yet recorded was photographed. Three smartphones were used, an iPhone 15 (4533 images), an iPhone 12 (241 images) and a Samsung Galaxy F22 (1003 images), each with its main and ultra wide cameras; one image carries no EXIF record. Photographs were taken under natural light, with flash on 700 images. Each class was photographed as isolated specimens (Single) and as market heaps (Bulk). Frames that were blurred, badly exposed, duplicated or dominated by unrelated objects were discarded by eye, and byte identical copies were removed by SHA-256 checksum. Labels are the Bangla trade names given by the sellers.
Data source location	• Institution: Karwan Bazar Fish Market; Rayerbazar Shutki Arot (Mohammadpur) • City/Town/Region: Dhaka • Country: Bangladesh • Latitude and Longitude (GPS coordinates) for collected samples: ○ Karwan Bazar: 23.751658131355093, 90.39428892788287 ○ Rayerbazar Shutki Arot: 23.74561656257414, 90.35870808008164
Data accessibility	Repository name: Science Data Bank (ScienceDB) Data identification number: https://doi.org/10.57760/sciencedb.25907 Direct URL to data: https://www.scidb.cn/en/detail?dataSetId=3745a925ab4b44c4aa0569d7fa79ab84 Instructions for accessing these data: The data are openly available under the CC BY 4.0 licence. This article describes version 5 of the deposit. Related code: scripts that derive the resized images, capture sessions, recommended split and checksums from the full resolution files are included in the deposit, in the folder code/, and at https://github.com/TanvirAnjumApurbo/BDryFish-tools
Related research article	None

## 1 Value of the Data

• BDryFish [1] records 22 dried fish trade categories as they are sold, photographed at the stalls of two wholesale markets in Dhaka with three smartphones, under natural light and with and without flash. It therefore tests image classification under the conditions that a practical recognition tool would meet.
• Every class is photographed both as isolated specimens (Single) and as market heaps (Bulk), so models can be trained on one presentation and tested on the other. This supports studies of transfer between presentation conditions, which a collection of isolated specimens alone cannot provide.
• A metadata table gives the capture time, device, lens, market and flash state of every image, with capture session identifiers at four thresholds and a recommended split that assigns whole sessions to one partition. Researchers can therefore keep burst frames from the same session together when benchmarking models and can study shifts across devices and capture days.
• The labels follow the trade names used in Bangladeshi markets rather than biological species. Pairs such as whole and sliced hilsa, or sun dried and fermented swamp barb, allow models to be tested on differences in processing and cut, which matter for food authentication and market inspection, although the classes are not a benchmark for species identification.
• The images are ready for image classification, since each class is a folder. Object detection or segmentation would require new annotation. Single images, with one specimen per frame, are the more practical starting point for bounding box annotation, whereas Bulk heaps of overlapping specimens would require labelling at the level of individual specimens.
• The collection documents products that are specific to the diet and culture of Bangladesh and are poorly represented in general food datasets such as Food-101 [2], which supports models adapted to South Asian markets.

## 2 Background

Bangladesh is often described as a land of rivers, and fish occupies a central place in its culture and economy. The saying Macche Bhate Bangali, which conveys that Bengalis live on fish and rice, reflects this dependence. Fresh fish spoils quickly, so preservation has long been necessary [3]. Sun drying is among the oldest and most widely practised methods, and the resulting product is known locally as Shutki. It is produced mainly in coastal regions and represents an important source of income for many households, particularly for women.

Beyond its cultural role, dried fish is economically significant. In 2020 to 2021, roughly twenty percent of the total catch of 4.6 million metric tons was converted into dried fish [4]. As dried fish grows in importance within diets and markets, reliable tools for identifying dried fish products become increasingly valuable. Such tools must recognise products as they are traded, and trade names in Bangladesh combine the species with the way the fish was processed and cut. The present dataset was compiled to support tools of this kind, and all images were recorded in authentic market environments so that they reflect how these products appear at the point of sale.

## 3 Data Description

The BDryFish dataset [1] comprises 5778 JPEG images of 22 dried fish trade categories sold in Bangladesh. The images were taken on 14 and 21 May 2025 with three smartphones (iPhone 15, iPhone 12 and Samsung Galaxy F22) at two wholesale markets in Dhaka and are released on Science Data Bank under the Creative Commons Attribution 4.0 International (CC BY 4.0) licence. Each class was photographed in two presentations. Single images show isolated specimens laid on a plain sheet, and Bulk images show the dense heap in which the product is displayed for sale, so that models can learn both isolated and heaped appearances. Table 1 gives the number of images in each class and presentation. Class sizes range from 210 images (Fermented Swamp Barb, 3.6% of the dataset) to 345 (Hilsa, 6.0%), a ratio of 1.64, and within the presentations from 98 to 207 Single images and from 98 to 169 Bulk images per class.

**Table 1:** Number of images in each class and presentation. Class folder names are the English names written without spaces, except Chouka, which has no established English name.

Sr. no	Bangla name	English name	No. of single images	No. of bulk images	Total images
1	Khalisha Shutki	Banded Gourami	98	161	259
2	Loitta Shutki	Bombay Duck	173	133	306
3	Chouka Shutki	Not established	207	112	319
4	Taka Chanda Shutki	Common Ponyfish	134	140	274
5	Chapa Shutki	Fermented Swamp Barb	112	98	210
6	Bashpata/Kajuli Shutki	Gangetic Ailia	139	104	243
7	Faisha Shutki	Gangetic Hairfin Anchovy	140	138	278
8	Boro Churi Shutki	Giant Largehead Hairtail	168	105	273
9	Gojar Shutki	Great Snakehead	126	102	228
10	Choto Chanda Shutki	Highfin Glassy Perchlet	107	169	276
11	Ilish Shutki	Hilsa	205	140	345
12	Surma Shutki	Indo-Pacific King Mackerel	118	110	228
13	Ghoinna Shutki	Kuria Labeo	135	106	241
14	Churi Shutki	Largehead Hairtail	126	165	291
15	Mola Shutki	Mola Carplet	114	167	281
16	Boiragi Shutki	Neglected Grenadier Anchovy	113	112	225
17	Poa Shutki	Pama Croaker	115	122	237
18	Rita Shutki	Rita	186	108	294
19	Kata Ilish Shutki	Slice Hilsa	119	118	237
20	Choto Chingri Shutki	Small Shrimp	110	106	216
21	Puti Shutki	Sun-Dried Swamp Barb	122	162	284
22	Chapila Shutki	White Sardinella	114	119	233
	**Total**		**2981**	**2797**	**5778**

Fig. 1 shows the directory structure. The deposit holds the same images in two resolutions, each in its own archive. The folder raw/ contains the full resolution files, and the folder resized640/ contains a copy of every image scaled so that its long side is 640 px. Both folders contain the same 22 class folders, which are named after the English names in Table 1 written without spaces (for example BandedGourami and Sun-DriedSwampBarb), except Chouka. Each class folder is divided into Single/ and Bulk/ subfolders. Files are named <Class>_<View>_NNNN.jpg, for example Hilsa_Single_0001.jpg, and are numbered in order of capture within each class and presentation, so the same filename denotes the same photograph in both resolutions. Beside them, the folders metadata/, splits/, checksums/ and code/ hold the files described below, which are distributed in a third archive. All three archives unpack into one top folder, BDryFish-v4/, which is named after the version in which the images last changed.

**Fig. 1:**
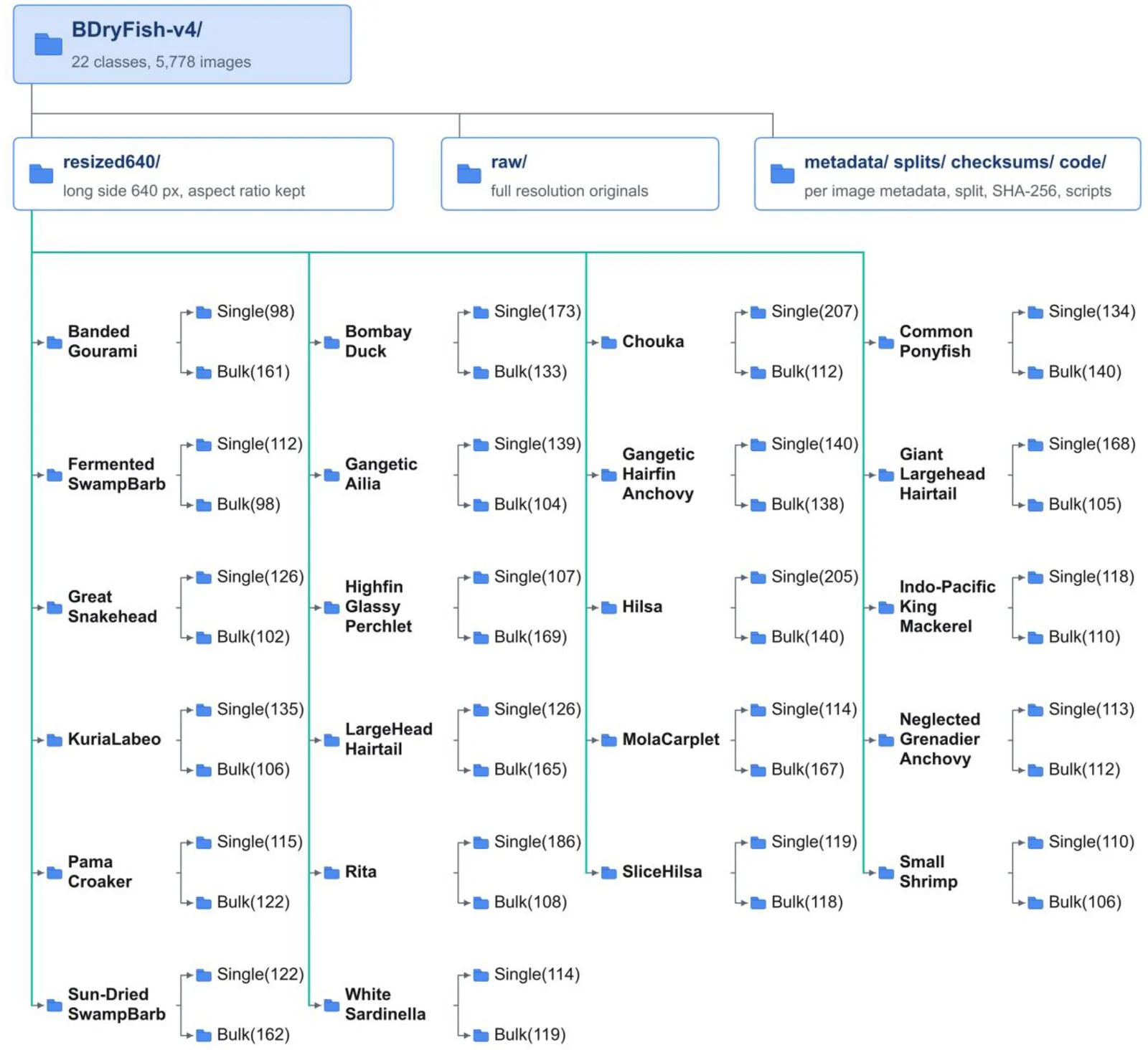
Directory structure of the BDryFish deposit. The raw/ and resized640/ folders contain the same 22 class folders, each divided into Single and Bulk subfolders, with the number of images given in parentheses. The folders metadata/, splits/, checksums/ and code/ hold the metadata for each image, the recommended split, the SHA-256 checksums and the scripts that reproduce them. All archives unpack into the top folder BDryFish-v4/. Class folder names are written without spaces, for example BandedGourami and Sun-DriedSwampBarb. The figure was drawn by a Python script, written with the help of Claude (Anthropic), which reads the folder names and image counts from the released files.

The files in raw/ are the photographs as retained after curation, ranging from 1.1 to 89.9 megapixels (long side 1372 to 10,948 px, median 12.2 megapixels). They are original JPEG files rather than unprocessed sensor (RAW) data, because photographs recorded in HEIC were converted to JPEG and 2531 frames (43.8%), identified by their dimensions, were cropped by hand, as described under Image Curation and Preprocessing. The files in resized640/ were derived from them by resampling alone, with the aspect ratio preserved and no padding or cropping, so they vary in size (788 distinct dimensions, with the short side between 124 and 640 px). Users who require square inputs should resize or pad the images at training time. The two resolutions differ in one further respect. In raw/, the orientation of 1482 images is recorded only in the EXIF Orientation tag, which image viewers apply automatically but many image loading libraries ignore, whereas in resized640/ the orientation has been applied to the pixels. Users of the full resolution files should therefore apply the EXIF orientation when loading them, for example with ImageOps.exif_transpose in Pillow. The full resolution archive occupies approximately 17.5 GB and the resized archive approximately 0.9 GB. All images are RGB JPEGs.

Table 2 gives the Bangla, English and scientific names of each class. The classes are trade categories, defined by the Bangla names under which sellers offer the products, and not taxonomic identifications. A trade name may combine a species with a processing state or cut, and the same name may be applied to more than one species. Two pairs of classes share a species and differ only in preparation. Ilish and Kata Ilish are both *Tenualosa ilisha*, whole and sliced, and Puti and Chapa were both matched to *Puntius chola*, sun dried and fermented. Churi and Boro Churi were both matched to *Trichiurus lepturus* and are sold as separate size grades, but the name Churi is also recorded for the Savalai hairtail, *Lepturacanthus savala* [5], so this pair may include a second species. For Boiragi, the English name follows *Coilia neglecta*, but the same local name is recorded for *C. ramcarati* [5], so the class is identified only to the genus *Coilia*. Chapila was matched to *Sardinella albella*, a marine species that carries this name [5], although the name also denotes the freshwater *Gudusia chapra* [6]. Choto Chingri is a small shrimp of the genus *Acetes*, a crustacean traded as shutki alongside fish. No established English or scientific name could be matched to Chouka, and the product may also be sold under other local names. The name Choukka is recorded for several marine herrings of the family Pristigasteridae, including *Ilisha melastoma, I. megaloptera* and *Pellona ditchela* [5], and the Chouka specimens, which are silvery, laterally compressed and resemble herrings in shape, are consistent with this group. The species could not be confirmed, so no scientific name is assigned. Chouka is retained as a class because the sellers named and sold it as a single, distinct product, which is the criterion applied to every class in the dataset. Users who need labels at the species level should merge or exclude classes using the processing_state, cut_form and same_species_as columns of metadata/classes.csv.

**Table 2:** Bangla trade names of the classes, with the matched English and scientific names and the relations between classes.

Bangla name	English name	Scientific name	Note
Khalisha	Banded Gourami	*Trichogaster fasciata*	
Loitta	Bombay Duck	*Harpadon nehereus*	
Chouka	Not established	Not assigned	Local name recorded for several marine herrings (Pristigasteridae)
Taka Chanda	Common Ponyfish	*Leiognathus equula*	
Chapa	Fermented Swamp Barb	*Puntius chola*	Fermented; same species as Puti
Bashpata/Kajuli	Gangetic Ailia	*Ailia coila*	
Faisha	Gangetic Hairfin Anchovy	*Setipinna phasa*	
Boro Churi	Giant Largehead Hairtail	*Trichiurus lepturus*	Larger size grade of Churi
Gojar	Great Snakehead	*Channa marulius*	
Choto Chanda	Highfin Glassy Perchlet	*Parambassis lala*	
Ilish	Hilsa	*Tenualosa ilisha*	Whole; same species as Kata Ilish
Surma	Indo-Pacific King Mackerel	*Scomberomorus guttatus*	
Ghoinna	Kuria Labeo	*Labeo gonius*	
Churi	Largehead Hairtail	*Trichiurus lepturus*	Name also used for *Lepturacanthus savala*
Mola	Mola Carplet	*Amblypharyngodon mola*	
Boiragi	Neglected Grenadier Anchovy	*Coilia* sp.	Name used for *C. neglecta* and *C. ramcarati*
Poa	Pama Croaker	*Otolithoides pama*	
Rita	Rita	*Rita rita*	
Kata Ilish	Slice Hilsa	*Tenualosa ilisha*	Sliced; same species as Ilish
Choto Chingri	Small Shrimp	*Acetes indicus*	Crustacean
Puti	Sun-Dried Swamp Barb	*Puntius chola*	Sun dried; same species as Chapa
Chapila	White Sardinella	*Sardinella albella*	Name also used for *Gudusia chapra*

Note. The Bangla trade names are the class labels. English and scientific names are the closest matches found online and checked against the Marine Biodiversity Portal of Bangladesh [5] and FishBase [6]; they are not verified taxonomic identifications.

Table 3 summarises the images by market, capture day, device, lens, flash and frame. The acquisition conditions are not balanced across classes. The Karwan Bazar images cover only Bombay Duck and Gangetic Hairfin Anchovy. Ten classes were photographed only on 21 May and one, Gangetic Ailia, only on 14 May. Every iPhone 12 and Galaxy F22 image was taken on 21 May, and five classes were photographed with the iPhone 15 alone. These imbalances matter for evaluation and are discussed under Limitations.

**Table 3:** Number of images by acquisition condition. The last column gives the number of classes with at least one image in that condition.

Condition	Level	Single	Bulk	Total (%)	Classes
Market	Karwan Bazar	280	229	509 (8.8)	2
	Rayerbazar Shutki Arot	2701	2568	5269 (91.2)	22
Capture day	14 May 2025	1464	987	2451 (42.4)	12
	21 May 2025	1517	1809	3326 (57.6)	21
Device	iPhone 15	2486	2047	4533 (78.5)	21
	iPhone 12	80	161	241 (4.2)	9
	Samsung Galaxy F22	415	588	1003 (17.4)	14
Lens	Main camera	2771	2150	4921 (85.2)	22
	Ultra wide camera	210	646	856 (14.8)	21
Flash	Fired	431	269	700 (12.1)	14
	Did not fire	2550	2527	5077 (87.9)	22
Frame	Native camera frame	1823	1424	3247 (56.2)	22
	Cropped in curation	1158	1373	2531 (43.8)	22
All		2981	2797	5778 (100)	22

Note. One Bulk image of Indo-Pacific King Mackerel carries no EXIF record, so its capture day, device, lens and flash state are unknown and it is omitted from those rows; its market is inferred from its position in capture order. The lens is inferred from the aperture recorded in EXIF, and a frame counts as cropped when its dimensions match no native output size of the recording phone.

The deposit also provides the metadata needed to reproduce, filter or split the dataset. The file metadata/images.csv has one row per image, giving the filename, class and presentation, the capture date and time with subsecond precision, the device, lens, market and flash state, the EXIF orientation of the full resolution file, the pixel dimensions in both resolutions, whether the full resolution frame matches a native output size of the phone, capture session identifiers at gaps of 2, 5, 10 and 30 s, the session and threshold used for the recommended split, the split assignment, and SHA-256 checksums of both files. The file metadata/classes.csv gives, for each class, the image counts, the Bangla, English and scientific names, the processing state, the cut form, any class of the same species and the notes of Table 2. The files metadata/removed_files.csv and metadata/duplicates.json list the files removed during curation, metadata/filename_map.csv maps the filenames of earlier versions to the current ones, splits/canonical_splits.csv holds the recommended split, and checksums/ holds SHA-256 checksums for every image file. The folder code/ contains the scripts that reproduce these files, and the resized images, from the full resolution files. A README, a datasheet and a changelog describe the deposit.

The deposit has been revised since its first release, and results obtained on different versions are not directly comparable. Version 1, deposited in 2025, contained 5875 files, and its resized copy was padded to 640 × 640 px with black bars and built without applying the EXIF orientation, so that 1482 resized images appeared rotated relative to their full resolution files. Because both the padding and the rotation varied with class, results obtained on version 1 may reflect these artefacts. Versions 2 and 3 changed the author list and added a README. Version 4, released in August 2026, removed one empty file and 96 byte identical duplicate copies, applied the EXIF orientation and removed the padding in the resized copy, renamed the files in order of capture and corrected the documentation. Version 5, released in October 2026, adds the metadata, split and checksum files described above and a corrected README, and leaves the images of version 4 unchanged. This article describes version 5.

## 4 Experimental Design, Materials and Methods

Fig. 2 presents an overview of the complete dataset construction workflow, spanning image acquisition, manual screening, preprocessing, labelling, and public release.

**Fig. 2:**
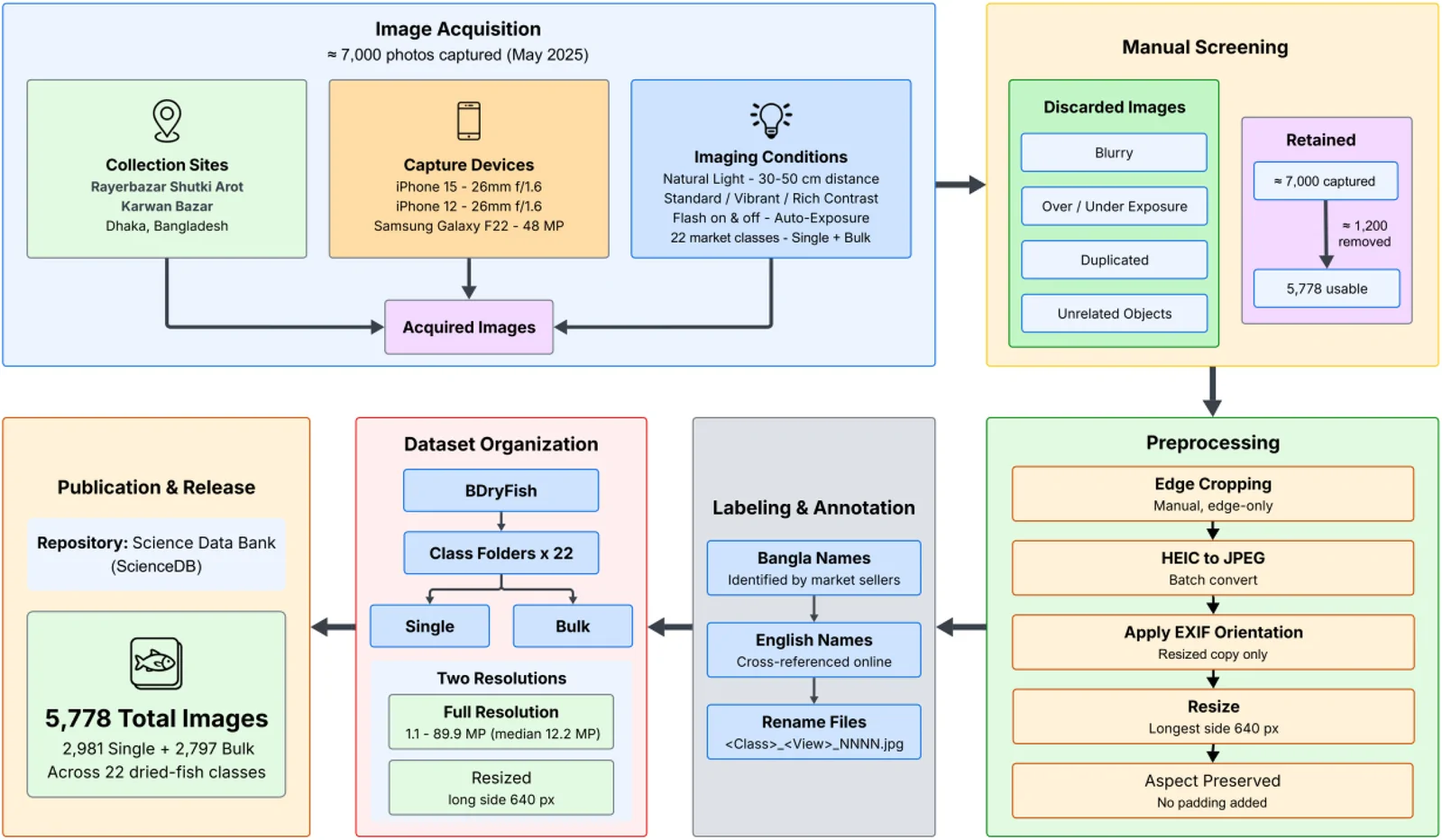
Workflow for constructing the BDryFish dataset, from image acquisition in the market to public release on Science Data Bank.

### 4.1 Image acquisition

Images were taken on two field days, 14 and 21 May 2025, at two wholesale markets in Dhaka, under natural light on sunny and cloudy days. Karwan Bazar is a wholesale market where traders buy stock to resell in local markets and where individual buyers can also purchase. The range of varieties there was limited, and the stalls of two vendors were photographed between 11:23 and 11:59 on 14 May, yielding 509 images of Bombay Duck and Gangetic Hairfin Anchovy. All other images were taken at Rayerbazar Shutki Arot in Mohammadpur, a larger wholesale depot that sells only in bulk and supplies markets such as Karwan Bazar. There, the authors visited every stall, roughly 24 in all, and photographed each variety that had not already been recorded. Varieties already recorded were generally not photographed again at other stalls, because the product looked alike from stall to stall. Ten classes were photographed only on 21 May and one only on 14 May (Table 3).

The primary device was an Apple iPhone 15, used with its main camera (26 mm equivalent, f/1.6) and its ultra wide camera (13 mm equivalent, f/2.4). It was supplemented by an iPhone 12 with the same pair of cameras and by a Samsung Galaxy F22 with its 48 MP main camera (f/2.0) and 8 MP ultra wide camera (f/2.2). Photographers held the phones approximately 30 to 50 cm from the specimens. On the iPhones, the Standard, Vibrant and Rich Contrast photographic styles were used, with and without flash, while the Galaxy F22 used its automatic settings. The photographic style was not recorded for each image, but the device, lens, flash state and capture time were preserved in the EXIF metadata. They are reported for every image in metadata/images.csv and summarised in Table 3. For Single images, specimens were laid on a plain sheet at the stall, and for Bulk images the heap was photographed as displayed. Photographs were usually taken in short bursts, so consecutive frames often show the same specimen or heap from almost the same viewpoint. The number of distinct specimens and heaps was not recorded, and the capture session defined below is therefore the countable unit of acquisition. Figs. 3 and 4 show one Single and one Bulk image of every class, illustrating the variation in background, illumination and composition.

**Fig. 3:**
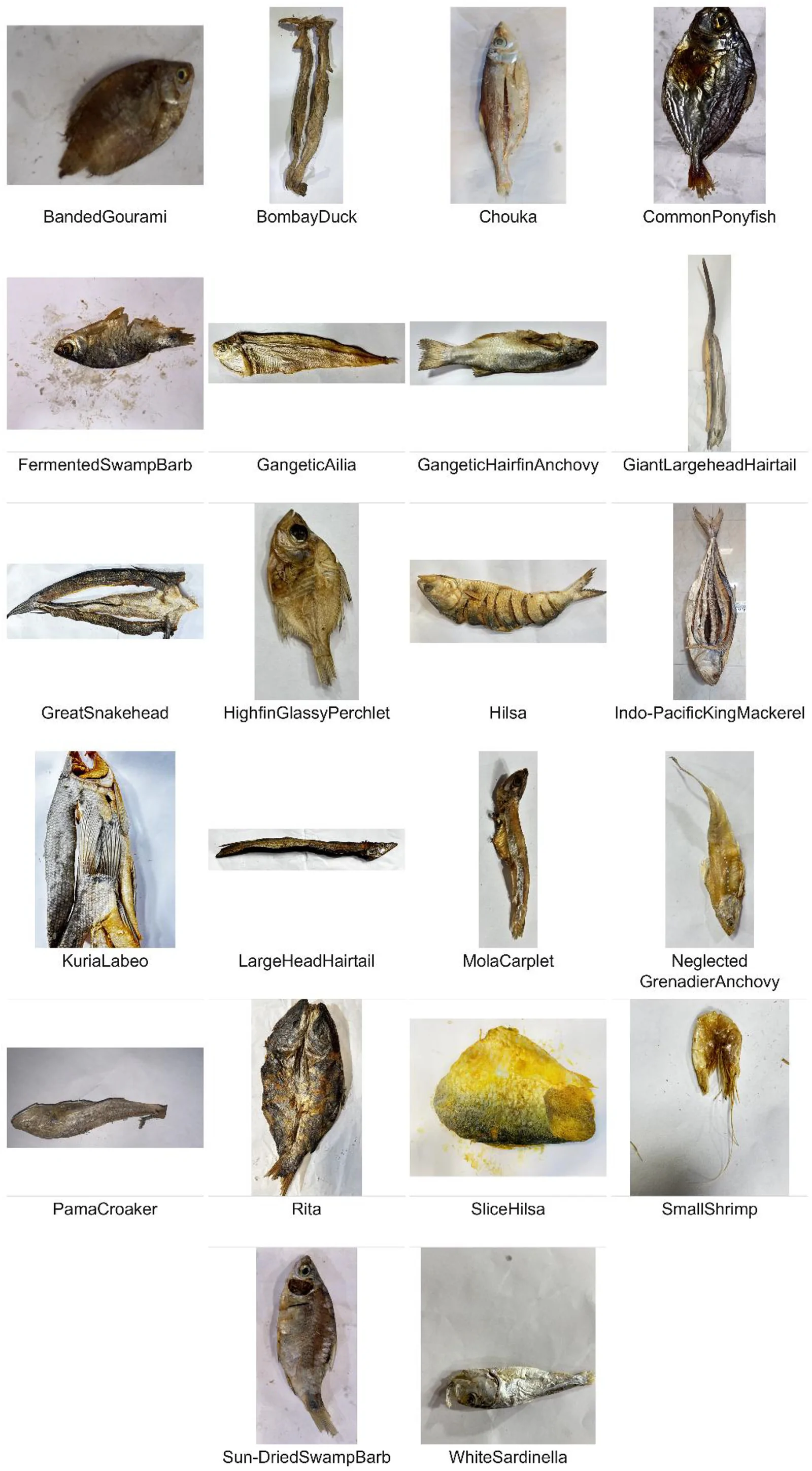
Collage of Single images from the 22 classes in the BDryFish dataset, highlighting individual specimen features.

**Fig. 4:**
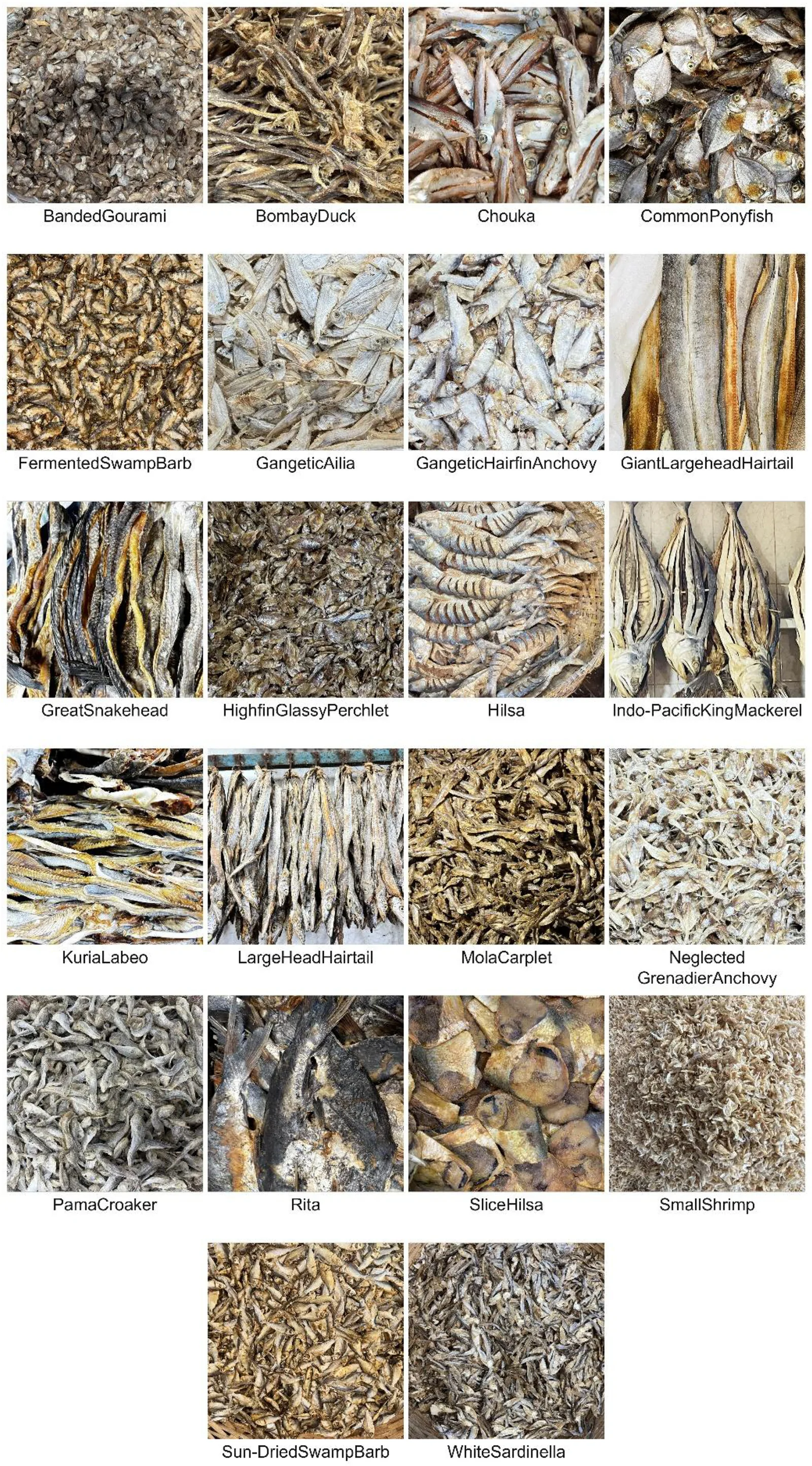
Collage of Bulk images from the 22 classes in the BDryFish dataset, showing typical market presentations.

### 4.2 Image curation and preprocessing

Photographs were first screened by eye. Frames that were blurred, severely over or under exposed, duplicated, or dominated by unrelated objects were discarded. Market conditions made many frames unusable, because there was rarely space to set a specimen down and photograph it at leisure. The number of frames discarded for each reason was not recorded. Of approximately 7000 photographs, 5875 were retained. An automated pass then computed a SHA-256 checksum for every file, and 96 byte identical copies in 87 groups were removed, keeping one copy of each; no group spanned two classes or presentations. One empty file of zero bytes was also removed, leaving 5778 images. Near identical frames from the same burst are distinct photographs and were retained, and they are grouped by the capture session identifiers described below.

Where a frame contained minor distractions near its edges, it was cropped by hand. In total, 2531 full resolution frames (43.8%) have dimensions that differ from every native output size of the recording phone, which indicates that they were cropped. HEIC files from the iPhones were converted to JPEG in batch with ImageMagick. The full resolution files were otherwise left unchanged. They retain their EXIF record of capture time and camera settings, although the software used for cropping or conversion removed some tags, such as the lens model, from 846 files, and no file contains GPS coordinates. Fifteen Galaxy F22 frames exceed the 48 MP of its main camera, reaching 89.9 MP, and their EXIF names the phone's camera application as the software that wrote them. To produce the resized copy, the EXIF orientation of each image was applied to its pixels and the tag reset, so that the resized images display in the same orientation as the full resolution files without relying on the tag. The long side was then scaled to 640 px with Lanczos resampling, and the image was saved as a JPEG at quality 95 with full chroma resolution (4:4:4), without padding, cropping or augmentation. No contrast adjustment or other enhancement was applied to either copy. Images were renamed <Class>_<View>_NNNN.jpg and numbered in order of capture within each class and presentation. No bounding boxes, key points or segmentation masks were produced. All processing used custom Python scripts with the open source Pillow and ImageMagick software. The scripts that derive the resized copy, the capture sessions, the recommended split and the checksums from the full resolution files are included in the deposit, in the folder code/, and in a public repository, https://github.com/TanvirAnjumApurbo/BDryFish-tools.

### 4.3 Labelling

Labels were assigned in the market. At every stall visited, the authors asked the seller to name each product and to say which fish it was, and recorded the Bangla trade name. Sellers at a wholesale market of this kind handle dried fish throughout their working lives and were treated as the most reliable available authority on these names. No conflicting names for the same product were recorded, and the names that the authors already knew agreed with those given by the sellers. The Bangla name is the class label. English and scientific names were matched afterwards by one author (K.F.A.), who searched each Bangla name online and compared the photographs and descriptions found with the specimens. Where a Bangla name returned little information, the candidate English name was searched and checked against the product. The matches were then checked against the Marine Biodiversity Portal of Bangladesh [5] and FishBase [6]. These names are therefore the best available correspondences and not independent taxonomic identifications. Trade names for dried fish are passed on orally by fishers and processors, vary between regions and dialects, and have no written standard, so a single name may cover more than one species, as Table 2 records.

### 4.4 Capture sessions and recommended evaluation

Because photographs were taken in bursts, many images have near twins taken seconds earlier or later. A random division of images into training and test sets would place such twins on both sides and overstate accuracy, so the release groups images into capture sessions. Within each class and presentation, images are ordered by capture time, and a new session begins whenever the gap to the previous frame exceeds a threshold, the device changes or the capture time is missing. Sessions are provided at thresholds of 2, 5, 10 and 30 s (Table 4). At 10 s, the 5778 images form 573 sessions, with a median of five frames and a maximum of 108.

**Table 4:** Capture sessions at each gap threshold, and the number of class and presentation cells whose recommended split uses that threshold.

Gap threshold (s)	Sessions	Sessions of one frame	Median frames per session	Largest session (frames)	Cells split at this threshold
2	2387	1244	1	31	2
5	1089	281	3	95	5
10	573	109	5	108	15
30	292	70	5	205	22

The recommended split assigns whole sessions to training, validation and test partitions. For each class and presentation, it uses the coarsest threshold that still yields at least four sessions, shuffles the sessions with a fixed seed, and places a quarter of them in the test partition and 15% of the remainder in the validation partition. Because some classes were photographed in a few long, continuous runs, the threshold was 30 s for 22 of the 44 class and presentation cells, 10 s for 15, 5 s for 5 and 2 s for 2. Frames taken within 2 s of each other in the same class and presentation are therefore never divided between partitions. At the 10 s threshold, however, 9.7% of Single and 26.5% of Bulk test images belong to a session that also contributes training images, and the proportions are higher at 30 s. The split contains 1790 training, 415 validation and 776 test images for Single, and 1667, 397 and 733 for Bulk, and every class is represented in every partition.

We recommend that users report results on this split and do not divide images at random. Stricter protocols can be built from the session identifiers, and the threshold used for each image is recorded so that any protocol can be reproduced. For evaluation across presentations, a model trained on one presentation should be tested on the whole of the other. When the aim is species identification, classes that share a species should be merged and Chouka excluded.

### 4.5 Comparative context

BDryFish sits within a growing collection of public image datasets of dried fish, several of which have appeared only recently (Table 5). Among Bangladeshi resources, DriedFishBD [7] provides 3288 images of eight small dried fish varieties photographed as single fish, bulk, head and tail in markets in Dhaka, BDDryFish [8] offers 1251 images of seven classes, and the Dry Fish Image Dataset of Hossain et al. [9] contains 3533 images of twelve classes photographed at Karwan Bazar. Beyond Bangladesh, the Indian dried seafood dataset of Paygude et al. [10] contains 8290 Single and Bulk images of five classes from a market in Pune, photographed against a standardised background. A further South Asian collection, SAR-DFD-2025 [11], is available, but its documentation names neither the country, the market nor the device used, so it is not included in Table 5. Against this landscape, BDryFish provides 22 classes, the broadest class coverage among these collections, although some of its classes share a species. It includes larger fish such as Hilsa and Indo-Pacific King Mackerel, and its images were taken at market stalls with three phones under varying light rather than against a standardised background. Among the collections in Table 5, it is the only one whose documentation reports the device and capture time of each image and provides a split that keeps capture sessions apart.

**Table 5:** Characteristics of public dried fish image datasets relevant to BDryFish. NR, not reported in the dataset documentation.

Characteristic	DriedFishBD [7]	BDDryFish [8]	Hossain et al. [9]	Paygude et al. [10]	BDryFish (this work) [1]
**Year**	2024	2025	2026	2024	2025 (version 5, 2026)
**Country**	Bangladesh	Bangladesh	Bangladesh	India	Bangladesh
**Collection site**	Markets in Dhaka	Unspecified (Bangladesh)	Karwan Bazar, Dhaka	Bombil Market, Pune	Karwan Bazar and Rayerbazar Shutki Arot, Dhaka
**Setting**	Market	NR	Market, natural light, plain and green backgrounds	Market, standardised background	Market stalls, natural light, with and without flash
**Devices**	Smartphone, number NR	NR	2 smartphones	2 smartphones	3 smartphones, main and ultra wide cameras
**Released image size**	1024 × 1024 px	NR	NR (scaled to 224 × 224 px for modelling)	1365 × 1024 px	1.1 to 89.9 MP originals and 640 px long side
**Classes**	8(a)	7	12	5	22 trade categories
**Images**	3288	1251	3533	8290	5778
**Presentations**	Single, bulk, head, tail	NR	NR	Single, bulk	Single, bulk
**Annotation reported**	NR	NR	Class labels (folders), species checked by two specialists	Class labels (folders)	Class labels (folders), seller trade names
**Metadata and split for each image**	NR	NR	NR	NR	Capture time, device, lens, market, flash; session identifiers; recommended split

(a) The DriedFishBD description states ten varieties but lists eight.

The value of the collection is further clarified by contrast with datasets of fresh fish. BD-Freshwater-Fish [12] targets fresh specimens in outdoor markets, whereas BDryFish targets processed, dried products. Because drying substantially alters texture, colour and shape, models trained on fresh fish cannot be assumed to transfer to dried products, which supports the need for a dedicated dried fish resource.

## Limitations

Several limitations should be acknowledged. They are grouped below by the use they affect, namely image classification in general, species recognition and the validity of benchmarks.

### Limitations affecting image classification

• **Lighting and variation.** Illumination at the stalls was natural and uneven, and flash fired on 700 images (12.1%), so some images contain deep shadows, reflections or overexposed regions that obscure texture. The flash state is recorded for each image, but the photographic style used on the iPhones is not.
• **Class imbalance.** The number of images per class ranges from 210 (Fermented Swamp Barb, 3.6% of the dataset) to 345 (Hilsa, 6.0%), a ratio of 1.64, and within presentations from 98 to 207 Single images and from 98 to 169 Bulk images per class (Table 1). Weighted losses or balanced sampling may be needed for even performance across classes.
• **Limited coverage.** All images come from two wholesale markets in Dhaka, were taken on two days in May 2025 and were recorded with three smartphone models. Seasonal variation, other regions of Bangladesh and South Asia, and other devices are not represented, and models may need retraining to generalise beyond these conditions.

### Limitations affecting species recognition

• **Trade categories, not species.** The 22 classes are trade categories. Ilish and Kata Ilish, and Puti and Chapa, share a species and differ in cut or preparation, and Churi and Boro Churi are sold as size grades of hairtail and may include two species. Accuracy on these pairs measures the recognition of processing, cut or size rather than species discrimination.
• **Unverified names.** English and scientific names were matched from online sources and have not been verified by a taxonomist. Several trade names are applied to more than one species, Boiragi is identified only to the genus *Coilia*, Chouka has no established scientific identity, and Choto Chingri is a crustacean. Some products are hard to identify once dried, and each label rests on the naming given by the sellers, so mislabelling is possible. The labels should not be relied on where species identity is critical, for example in regulatory or food safety decisions.

### Limitations affecting benchmark validity

• **Burst capture and near duplicates.** Many frames were taken seconds apart, so a random split places near identical images on both sides and overstates accuracy. The recommended split keeps sessions apart at the thresholds listed in Table 4, but at 10 s, 9.7% of Single and 26.5% of Bulk test images still share a session with training images, because some classes were photographed in a few long runs.
• **Acquisition conditions confounded with class.** Market, capture day and device are unevenly distributed across classes (Table 3). The Karwan Bazar images cover only two classes, ten classes were photographed only on 21 May, when all iPhone 12 and Galaxy F22 images were taken, and five classes were photographed with a single phone. A model may therefore exploit cues of device or capture day, and evaluations that hold out a day or a device change the class composition as well as the acquisition conditions.
• **Manual cropping.** About 44% of the full resolution frames appear to have been cropped by hand during curation, so framing and image size reflect curatorial choices as well as the scene.

Future expansions could add images from other seasons, regions and devices, photograph each variety at several stalls with records of individual specimens, and obtain independent taxonomic verification. Even so, BDryFish offers a documented starting point for recognising dried fish as it is traded.

## Ethics Statement

The authors confirm that they have read and complied with the ethical requirements for publication in Data in Brief. This work does not involve human subjects, animal experiments, or data collected from social media platforms.

All images in the BDryFish dataset were collected in open-air markets with the knowledge and verbal consent of the vendors. Photographs were taken solely of dried fish products for the purpose of dataset development. Human subjects (e.g., vendors or shoppers) were not the focus and were intentionally kept out of frame. No identifiable personal information, such as faces, ID cards, or other sensitive data, is present in the dataset. Every released image file was scanned for a GPS metadata block, and none was found; the market coordinates given in the specifications table describe the collection sites, not individual photographs.

## Declaration of Generative AI and AI-assisted Technologies in the Manuscript Preparation Process

During the preparation of this work, the authors used Claude by Anthropic and GPT by OpenAI to improve the clarity, grammar, and organisation of the manuscript, and Claude by Anthropic to help create Fig. 1, which is drawn by a Python script from the released files. After using these tools, the authors reviewed and edited the content as needed and take full responsibility for the content of the published article.

## CRediT authorship contribution statement

**Tanvir Anjum Apurbo:** Writing – original draft, Project administration. **Kh Faysal Ahammed:** Validation, Investigation, Resources, Writing – original draft, Visualization. **Md. Abdullah:** Writing – review & editing, Investigation. **Mohosinin Islam:** Data curation, Investigation. **Md. Habibullah Belali:** Conceptualization, Supervision.
